# Complete Genome Sequence of a Genomovirus Associated with Common Bean Plant Leaves in Brazil

**DOI:** 10.1128/genomeA.01247-16

**Published:** 2016-11-10

**Authors:** Natalia Silva Lamas, Rafaela Salgado Fontenele, Fernando Lucas Melo, Antonio Felix Costa, Arvind Varsani, Simone Graça Ribeiro

**Affiliations:** a Embrapa Recursos Genéticos e Biotecnologia, Brasília, Brazil; b Universidade de Brasília, Brasília, Brazil; c Instituto Agronômico de Pernambuco, Recife, Pernambuco, Brazil; d Structural Biology Research Unit, Department of Clinical Laboratory Sciences, University of Cape Town, Cape Town, South Africa; e Center of Fundamental and Applied Microbiomics, The Biodesign Institute and School of Life Sciences, Arizona State University, Tempe, Arizona, USA

## Abstract

A new genomovirus has been identified in three common bean plants in Brazil. This virus has a circular genome of 2,220 nucleotides and 3 major open reading frames. It shares 80.7% genome-wide pairwise identity with a genomovirus recovered from Tongan fruit bat guano.

## GENOME ANNOUNCEMENT

*Genomoviridae* is a recently established family of circular replication–associated protein (Rep)–encoding single-stranded (CRESS) DNA viruses (1). The family currently has one genus, *Gemycircularvirus*, with a single recognized species, *Sclerotinia gemycircularvirus* 1. There are more than 100 genomoviruses that have been identified in various organisms and environmental samples summarized in Krupovic et al. (1). Within a Brazilian context, genomoviruses have been identified in human feces, river, and wastewater samples (2, 3). Here, we report the recovery of genomovirus genomes from bean plants (*Phaseolus vulgaris*) showing viral-like symptoms, such as leaf curling, mosaic, and stunting, which were collected in Arcoverde, Pernambuco state, Brazil. 

Total DNA was extracted from these leaf samples, and circular viral DNA was enriched by rolling circle amplification (RCA) using Phi29 DNA polymerase (NEB, USA) (4). The RCA amplicons from all plants were pooled and sequenced using a GS-FLX + 454 sequencing platform at Macrogen Inc. (South Korea). The reads were analyzed for sequence similarities to CRESS DNA viral sequences. A 123-nucleotide (nt) read with similarities to genomovirus sequences was identified, and back-to-back primers (GemyNew forward primer 5′ TCGAATCCATCGGGTATTGTGGG 3′ and GemyNew reverse primer 5′ ATTCGATACGGACGGCCTTATC 3′) were designed to recover the full viral genomes from individual samples by inverse PCR with Phusion DNA polymerase (NEB). Amplicons of ~2.2 kb were obtained from three samples, which were gel-purified (GE Healthcare, USA), cloned into PCRII-TOPO-TA vector (Thermo Fisher Scientific Inc., USA) and Sanger-sequenced at Macrogen Inc. by primer walking. The complete circular genomes of 2,220 nt in length have three large bidirectionally oriented open reading frames (ORFs). One ORF in the virion sense encodes a putative viral capsid protein and, in the antisense, two other proteins are encoded, Rep and RepA. The new sequences have been tentatively named common bean–associated gemycircularvirus (CBaGmV). The CBaGmV genome sequences share 80.7% genome-wide nucleotide pairwise identity with a genomovirus from Pacific flying fox feces (PfffaCV-6; accession no. KT732799). The CBaGmV CP, Rep, and RepA amino acid sequences are. respectively, 76%, 88%, and 88% similar to the homologous proteins of PfffaCV-6 (5).

### Accession number(s).

The complete genomes of CBaGmV described in this report have been deposited in GenBank under accession numbers KX434768 to KX434770.
